# Individual Differences in Brain Responses: New Opportunities for Tailoring Health Communication Campaigns

**DOI:** 10.3389/fnhum.2020.565973

**Published:** 2020-12-03

**Authors:** Richard Huskey, Benjamin O. Turner, René Weber

**Affiliations:** ^1^Cognitive Communication Science Lab – C^2^ Lab, Center for Mind and Brain, Department of Communication, University of California, Davis, Davis, CA, United States; ^2^Wee Kim Wee School of Communication and Information, Nanyang Technological University, Singapore, Singapore; ^3^Media Neuroscience Lab, Department of Communication, University of California, Santa Barbara, Santa Barbara, CA, United States

**Keywords:** prevention neuroscience, persuasion neuroscience, individual differences, public service announcements, health campaigns, media neuroscience

## Abstract

Prevention neuroscience investigates the brain basis of attitude and behavior change. Over the years, an increasingly structurally and functionally resolved “persuasion network” has emerged. However, current studies have only identified a small handful of neural structures that are commonly recruited during persuasive message processing, and the extent to which these (and other) structures are sensitive to numerous individual difference factors remains largely unknown. In this project we apply a multi-dimensional similarity-based individual differences analysis to explore which individual factors—including characteristics of messages and target audiences—drive patterns of brain activity to be more or less similar across individuals encountering the same anti-drug public service announcements (PSAs). We demonstrate that several ensembles of brain regions show response patterns that are driven by a variety of unique factors. These results are discussed in terms of their implications for neural models of persuasion, prevention neuroscience and message tailoring, and methodological implications for future research.

## Introduction

The field of prevention neuroscience is organized around understanding the neural mechanisms that underpin health attitude and behavior change (for a review, see Hall et al., 2018). Numerous studies demonstrate that neural activity in response to persuasive messages can accurately predict health attitudes and behavior change, and that adding neural activity to traditional self-report measures results in substantially better prediction models (Chua et al., 2011; Falk et al., 2015; Weber et al., 2015a). These findings have led to several important advances. For instance, a constellation of structures in the persuasion network (Falk et al., 2010, see also section “Persuasion Neuroscience”) has been shown to systematically vary in response to different persuasive messages (Kaye et al., 2016), and research demonstrates that this variation is useful for accurately characterizing which health communication campaigns are most likely to succeed (Falk et al., 2016).

However, despite considerable progress, this research largely depends on aggregating neural responses across individuals and focusing on group-level results. This approach has undoubtedly led to rapid and widespread advances in linking brain structure to psychological function. However, group-level aggregation does have two shortcomings^1^. First, it has the potential to muddy the interpretation of results on a neuroanatomical level. And second, it misses out on the potential power of using neuroimaging to improve health message tailoring. For example, regarding the first shortcoming, research shows that high drug-risk individuals, characterized by high issue involvement, exhibit qualitatively different patterns of brain activation and functional connectivity (suggestive of counter-arguing against putatively highly persuasive messages) than low drug-risk individuals (Weber et al., 2015a; Huskey et al., 2017). In both cases, ignoring the individual difference dimension of drug-risk would have led to a hybrid pattern of results that likely did not occur in any of these two groups. This logic can be extended to all individuals in a neuroimaging study—ignoring theoretically relevant differences on the individual level can lead to a hybrid pattern of results that likely did not occur in any individual (Haxby et al., 2011; Davison et al., 2016). Regarding the second limitation, particularly in the context of health message campaigns, maximizing the effectiveness of any particular message requires considering not only the features of the message itself, but also how these features align with the viewer on a number of different dimensions (Rimer and Kreuter, 2006). In short, tailored messages are more effective than untailored messages (Noar et al., 2007; Lustria et al., 2013).

Of course, heterogeneity in neural response can be partially investigated by conducting moderation analyses (Schmälzle and Meshi, 2020). But even this sort of analysis tells only part of the story. Any approach that treats individuals as interchangeable can only partially contribute to our understanding of tailoring effects. To overcome these concerns, our study applied a multi-dimensional *similarity-based* individual differences analysis (Miller et al., 2002, 2009, 2012; van Baar et al., 2019; Turner, 2020) to explore which individual factors—including characteristics of messages and target audiences—drive patterns of brain activity to be more or less similar across individuals encountering the same anti-drug public service announcements (PSAs). We demonstrate that several ensembles of brain regions show response patterns that are driven by a variety of unique factors. These results are discussed in terms of their implications for neural models of persuasion, prevention neuroscience and message tailoring, and methodological implications for future research. We conclude with a discussion of future directions for incorporating the individual as an entity of interest in persuasion neuroscience research.

### Persuasion Neuroscience

The last decade has seen substantial progress in our understanding of the persuasion network (Falk et al., 2010)—that is, the collection of brain regions that are activated while individuals are encountering persuasive messages (for a recent critical review, see Cacioppo et al., 2017). Research in this domain has led to a number of advances, including an increasingly resolved map of the network’s putative constituent regions (Falk et al., 2010; Kaye et al., 2016), the factors to which they are sensitive (Falk and Scholz, 2018), how they represent persuasive messages (Pegors et al., 2017), their interconnections (Ramsay et al., 2013; Huskey et al., 2017; Cooper et al., 2018), and their neural similarities in persuasive message processing across audience members (Imhof et al., 2017, 2020). In addition to shedding light on theoretical debates (e.g., Weber et al., 2015a), these results in the neural domain have been shown to offer real-world utility by improving predictions of subsequent behavior above and beyond traditional measures (for a review, see Berkman and Falk, 2013).

In parallel with this work toward characterizing the typical persuasion network, persuasion scholars have made substantial progress in the past two decades in understanding the effects of message tailoring. All things being equal, tailored messages (messages that align source, message, receiver, and context factors) are more persuasive compared to untailored messages (Rimer and Kreuter, 2006; Noar et al., 2007; Lustria et al., 2013). Neuroimaging research demonstrates that a number of regions of interest (ROIs) are more active when processing tailored (as compared to untailored) persuasive messages, including: the medial prefrontal cortex (MPFC), precuneus, and posterior cingulate (Chua et al., 2009). Similarly, neural responses to tailored messages, particularly in the MPFC, are predictive of behavior change (Chua et al., 2011). Current models of persuasion heavily implicate the ventral MPFC (vMPFC) as well as the ventral striatum (VS) in persuasion (Falk and Scholz, 2018), and the MPFC has been targeted as a candidate region of interest for examining the influence of message tailoring (Tompson et al., 2015).

Despite this progress, a number of unanswered questions remain (Cacioppo et al., 2017). First among them, pattern of interest (POI) analyses demonstrate that whole-brain responses to persuasive messages provide unique information above and beyond that gained by ROI-based analyses (Doré et al., 2019). This aligns with evidence in the behavioral literature that identical persuasive outcomes (e.g., attitude changes, behavioral intentions, or actual behavior changes) can be observed with different sets of underlying cognitive processes (for seminal research on this issue, see Petty and Cacioppo, 1984; also see Weber et al., 2013), and suggests potential structural updates to the persuasion network. Second, many of the aforementioned findings largely depend on aggregating across individuals and focusing on group-level results. This approach largely ignores individual variance in persuasive message processing by treating individual-level variance as noise. A complementary approach, one that treats individual-level variance as a signal, has the potential to not only expand our understanding of the neural basis of persuasion, but to also improve our capacity for tailoring effective persuasive messages.

### The Individual Differences Approach

There is an increasing understanding that individuals vary and that any given behavioral response might be driven by a variety of individually different neural processes (Marder, 2011; Krakauer et al., 2017). In the present study, we examine this by applying some of the techniques from individual differences research in the neural domain (Miller et al., 2002, 2009, 2012; Dubois and Adolphs, 2016) to a study of anti-drug public service announcement (PSA) viewing. Briefly, an individual differences analysis is one that seeks to link neural responses to relevant individual characteristics (for an overview of the method, see Turner et al., 2019). Some of the studies mentioned above, particularly the ones that adopt a brain-as-predictor approach (Berkman and Falk, 2013), already meet the rather expansive criteria of this definition.

We expand on this approach with an analytical procedure that is conceptually similar to representational similarity analysis (Kriegeskorte et al., 2008). This multi-dimensional similarity-based approach (see Miller et al., 2009, 2012) considers how pairwise similarity in participant neural responses can be explained by pairwise similarities in participant individual difference measures. This procedure, which is an extension of multivariate distance matrix regression (Anderson, 2001), is beneficial because it provides researchers with a principled method for identifying how much variance a given individual difference measure explains in a neural response while also controlling for other observed and unobserved individual difference measures (Turner et al., 2019).

The rationale for conducting such an analysis is straightforward. A number of studies have shown that even simple cognitive tasks such as episodic, semantic, and working memory protocols (Miller et al., 2002, 2009, 2012), attentional, resting, and multi-modal memory tasks (Davison et al., 2016), and natural speech processing (Huth et al., 2016) show considerable individual differences in neural response. A common characteristic among these studies is variation in neural response within participants was low (both across task type and over time) while between participant variation was high. A common interpretation of this result is that participants use a number of different cognitive strategies to perform the same behavioral task (for an extended discussion, see Krakauer et al., 2017). By comparison, processing persuasive messages such as the PSAs used in this study is a complex task and requires an even higher number of cognitive processes (e.g., audiovisual, speech/language, self/other-references, logical/causal reasoning, emotions). Thus, we should expect that processing persuasive messages corresponds to an even higher level of variation in neural responses between participants (see e.g., Hawco et al., 2020).

With this rationale in mind, a central question in this paper is: what does variation in neural responses between participants tell us? A number of prominent papers have forcefully argued that these individual differences provide important signal that helps us better map structure to function, identify and diagnose pathology, and (crucially for this study) identify the sources of variability (Van Horn et al., 2008; Gabrieli et al., 2015). Recent evidence demonstrates that naturalistic tasks such as watching audiovisual stimuli (e.g., the PSAs used in this study) are well suited for examining sources of individual variability (for a review, see Eickhoff et al., 2020).

Our study uses an individual differences analysis to address two aims. The first is to better understand the mapping between structure and function for persuasive message processing. The second is to understand sources of individual variation in persuasive message processing with an aim toward identifying potential avenues for message tailoring. Together, these results will help us understand what individual difference characteristics drive neural responses to persuasive messages, which should provide information that informs future attempts at message tailoring while also identifying potential ROIs for future brain-as-predictor based investigations.

### The Present Study

In this study, we evaluate participant responses to a number of 30 s anti-drug PSAs. These PSAs systematically vary in terms of their argument strength (AS) as well as their levels of arousal (message sensation value; MSV), two dimensions that have been demonstrated to influence message persuasiveness (see e.g., Weber et al., 2013). We expose participants who are at either high- or low-risk for drug use to each of these messages. Importantly, drug-risk has been shown to interact with message characteristics, specifically the interaction between MSV and AS, and result in different behavioral (Weber et al., 2013) and neural (Weber et al., 2015a; Huskey et al., 2017) responses. Moreover a large body of theoretical research investigating the Elaboration Likelihood Model demonstrates that the interaction between MSV and AS influences persuasive message processing (Petty and Cacioppo, 1986). Therefore, we will focus our inquiry on this interaction term. This procedure is particularly suitable for the present study as it generates considerable variability in individual-level responses. By systematically evaluating this variability, we evaluate the extent to which a number of *a priori* and exploratory ROIs are sensitive to individual difference measures.

## Materials and Methods

### Previous Reporting and General Overview

This manuscript uses fMRI data previously reported in Weber et al. (2015a). This earlier analysis relied on the classic general linear model to characterize differences in group-level activation maps for high- compared to low-drug use risk participants. By comparison, the analysis and results reported in this manuscript adopt a multi-dimensional similarity-based individual differences approach to examine unique neural activation patterns at the individual participant level. The present study also includes several new individual difference profiling variables that have not been previously reported on. Together, this new analytical approach coupled with the inclusion of new individual difference variables allows us to examine new questions beyond what has been previously reported. Nonetheless, because the analyses conducted for the current study repurpose data originally collected to test related but distinct hypotheses, the results should be seen as a starting point for inductive hypothesis generation and future research in the deductive mode.

#### fMRI Data Acquisition

Data were acquired on a Siemens Magnetom TIM Trio scanner with a 3-Tesla magnetic field strength. An 8-channel phased-array headcoil was used during acquisition. T2^∗^-weighted images were acquired using a single-short echo planar gradient sequence (TR = 2,000 ms, TE = 27.2 ms, FA = 77 degrees, FOV = 22 × 22 cm^3^). Forty interleaved slices were acquired parallel to the AC-PC plane (slice thickness = 3 mm, 0 mm gap, 64 × 64 matrix). A high-resolution T1-weighted sagittal sequence (TR = 1,620 ms, TE = 3.87 ms, FOC 250 mm, voxel resolution 1 mm isotropic) was also collected.

#### fMRI Pre-processing

The fMRI data used in this study were pre-processed using FEAT (fMRI Expert Analysis Tool v6.0) from the Oxford Center for Functional MRI of the Brain (FMRIB) Software Library (FSL v5.0). The pre-processing pipeline followed standard conventions for cleaning fMRI data (Weber et al., 2015b). The data were motion corrected using FSL’s Motion Correction FMRIB Linear Registration Tool (Jenkinson et al., 2002) and the in-brain data were masked using FSL’s Brain Extraction Toolkit (BET; Smith, 2002). Data were highpass filtered (σ = 59.5 s) and grand-mean intensity normalized. FSL’s FLIRT utility (Jenkinson and Smith, 2001; Jenkinson et al., 2002) was used to align participant functional data to a high-resolution T1-weighted structural scan. Finally, the data were resliced to 5 mm isotropic voxels using FLIRT with nearest-neighbor interpolation.

#### Participants and Experimental Procedure

Twenty-eight participants were characterized along a number of individual difference dimensions (see section “Intrinsic Measures” below). Participants viewed 32 anti-marijuana PSAs (for complete details, see Kang et al., 2006) made available by the anti-drug PSA archive at the University of Pennsylvania, Annenberg School for Communication. The stimulus PSAs were extensively pre-tested to constitute a fully crossed design of low and high message sensation value (MSV) with low and high argument strength (AS; for more details regarding the rationale, operationalization, and interpretation of these variables, see Weber et al., 2013). Each PSA lasted 30 s, and PSAs were interspersed with control clips (also 30 s in length) where the video was scrambled (thus destroying message meaning while preserving luminosity and auditory amplitude), and with blank 10 s intervals between each PSA. In addition, participants completed a number of individual difference measures (see section “Participant PSA Evaluations” below) and evaluated each PSA on various dimensions. We examined the relationships between these individual difference measures, PSA evaluation measures, and patterns of brain activity across a set of regions. These regions were selected *a priori* from previous findings in the literature and *a posteriori* from functional maps using the interaction of MSV and AS as contrast (see Table 1 and section “Region of Interest Analysis” below).

**TABLE 1:** A list of definitions for key terms and acronyms in the manuscript.

Term/Acronym	Definition
**Regression model**	
Intrinsic measures	Participants were evaluated on their risk for using marijuana, sensation seeking, and overall compliance with the task (see “ID Variables” below).
PSA measures	PSA-related measures considered participants’ responses to the videos along a number of dimensions, including thought valence, ad liking, positivity/negativity, as well as pAS and pMSV (see “ID Variables” below).
Neural measures	Neural measures include structural and functional measures of similarity, at both the whole-brain and ROI level (see “ID Variables” below).
Shared variance	Captures the (shared) variance explained in the full model that is not explained by any unique variable or set of variables — the variance that is explained in the subspace defined by the covariance amongst the other predictors.
Unexplained	A set of participant-specific intercepts (subints)—in other words, unexplained by the set of variables we have included, but in principle explainable on the basis of other (unknown) individual difference factors.
**ROIs**	
*A priori*	ROIs that are derived from the past literature (Table 2, IDs 1–4).
Exploratory	The 10 peaks that demonstrated the most between-participant variability in the MSVxAS contrast (see Weber et al., 2015a; Table 2, IDs 5–14).
Confirmatory	These peaks come from Table 1 of Weber et al. (2015a), from the MSVxAS contrast for the high drug-risk group (Table 2, IDs 15–23).
**Individual difference (ID) variables**	
MJrisk	“Risk for marijuana use scale” by Cappella et al. (2003).
SSscore	Sensation seeking is defined as the tendency to seek out novel, complex, or exciting situations and stimuli (Zuckerman, 1994).
Compliance	Overall involvement and compliance with the task.
pAS	Perceived argument strength (Zhao et al., 2011).
pMSV	Perceived message sensation value (Palmgreen et al., 2009).
pos/neg	14 items collapsed into two measures by averaging three positive (pos: “good,” “happy,” “inspired”) and seven negative (neg: “sad,” “afraid,” “bad,” “guilty,” “angry,” “disgusted,” “sympathetic”) items.
AdLike	Ad liking was measured with a single seven-point Likert item asking the degree to which participants liked each PSA (Kang et al., 2006).
ThVal	Thought valence—degree to which positive or negative thoughts about the message predominate (Petty and Cacioppo, 1986; Kang et al., 2006).
ROIneur	SPMs of the activity related to two different control conditions shown in the PSA experiment (scrambled videos as well as a blank screen).
wbFxnSim	Whole-brain functional similarity, which we treat as a control.

#### Intrinsic Measures

A number of theoretically relevant measures intrinsic to participants were collected via self-report. Specifically, participants were evaluated on their risk for using marijuana, sensation seeking, and overall compliance with the task.

Drug use risk was measured with the “risk for marijuana use scale” by Cappella et al. (2003). Sensation seeking is defined as the tendency to seek out novel, complex, or exciting situations and stimuli. The construct was measured using a four-item scale derived from Zuckerman (1994). The items were “I like to explore strange places,” “I like to do frightening things,” “I like new and exciting experiences even if this breaks rules,” and “I prefer exciting and unpredictable friends” (1–5, strongly disagree, disagree, neutral, agree, strongly agree). Overall involvement and compliance with the task was measured with a self-made four-item scale (1–5, strongly disagree, disagree, neutral, agree, strongly agree). The items were “The study was fun,” “The study was interesting,” “I would recommend friends doing the study,” “I enjoyed being part of this study.”

#### Participant PSA Evaluations

In addition to these intrinsic measures, participants completed a number of self-reported perceptions of each PSA. Perceived message sensation value (pMSV; Palmgreen et al., 2009) was measured to evaluate participant perceptions of PSA emotional arousal, dramatic impact, and novelty. Perceived argument strength (pAS; Zhao et al., 2011) measured how strong/weak participants thought a PSA was. Ad liking (AdLike) was measured with a single seven-point Likert item asking the degree to which participants liked each PSA (Kang et al., 2006). Another set of questions, each rated on a 1–4 Likert scale, asked participants to agree with 14 emotion statements of the form “Please indicate how much this ad made you feel…” These 14 items were collapsed into two measures by averaging three positive (pos: “good,” “happy,” “inspired”) and seven negative (neg: “sad,” “afraid,” “bad,” “guilty,” “angry,” “disgusted,” “sympathetic”) items. Finally, thought valence (ThVal)—the degree to which positive or negative thoughts about the message predominate following message receipt—was measured as the difference score between two 7-point Likert items regarding the PSA generating thoughts about wanting to try marijuana and generating thoughts about staying away from marijuana (Petty and Cacioppo, 1986; Kang et al., 2006). Each of these ratings was assessed after a second viewing that took place outside of the scanner. For the analyses described below, missing values for any given scale or question were simply replaced by the mean across all non-missing responses for that scale/question.

#### Open Science Practices

In accordance with recent calls to make scientific studies more transparent and reproducible (Poldrack et al., 2017; Dienlin et al., 2020), the preprocessed data and analysis code for the results reported in this submission are available in a public repository on the Open Science Framework^2^.

### Region of Interest Analysis

Our goal was to explore how message and audience characteristics relate to individual differences in patterns of brain activity across 23 regions of interest, which comprised a set of *a priori* regions within the persuasion network as well as those defined *a posteriori* on the basis of whole-brain analyses. Each of these steps—defining ROIs, defining individual difference measures, and relating brain activity to these measures—is described in turn below.

**FIGURE 1:**
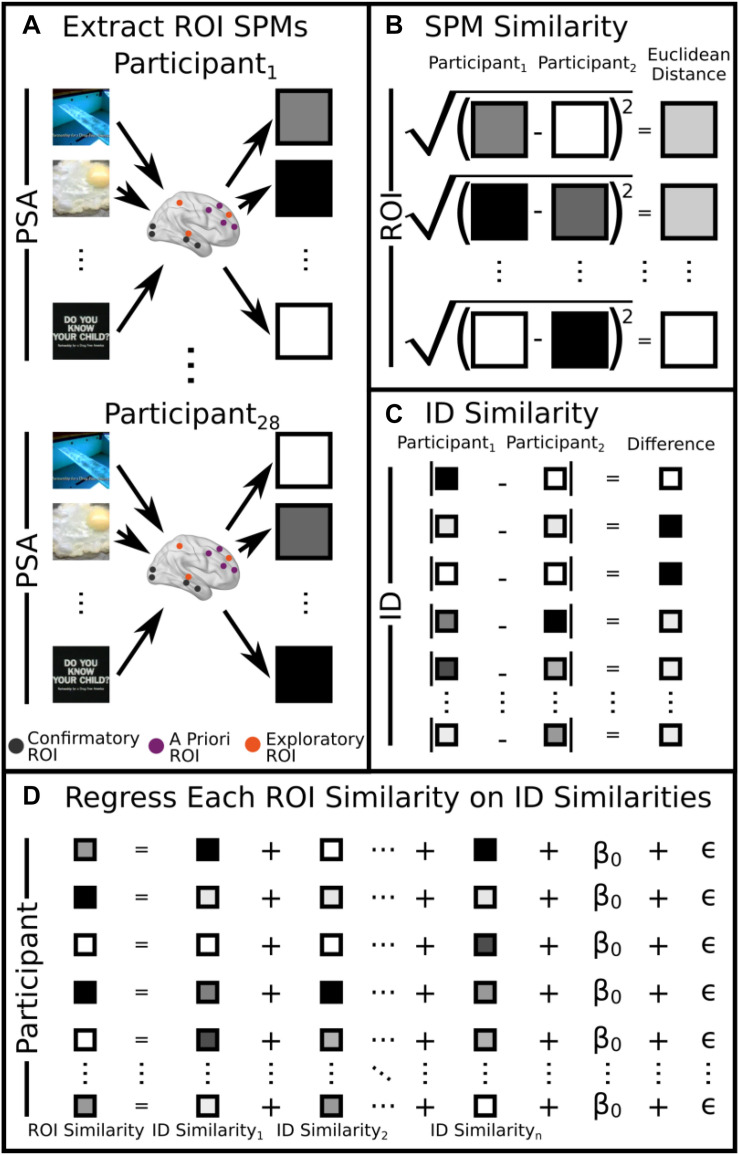
Visual schematic of the analysis. **(A)** Neural SPMs were extracted for the MSVxAS interaction term for confirmatory, *a priori*, and exploratory ROIs. **(B)** Pairwise similarity was calculated by computing the Euclidean distance between SPMs for each participant for each ROI (here, we show this procedure for the pairwise comparison between participant 1 and participant 2). **(C)** For individual difference variables, pairwise similarity was calculated by taking the absolute value of the difference between participant pairs (again, we show the pairwise comparison for participant 1 and participant 2). **(D)** Pairwise similarities for each ROI were regressed on the pairwise similarities for each individual difference measure. A round-robin procedure was used to extract *R*^2^ for each regressor.

Regions of interest were derived from three sources: (1) *A priori* ROIs: these are ROIs that are derived from the past literature (see Table 2, IDs 1–4). (2) Exploratory ROIs: these reflected the 10 peaks that demonstrated the most between-participant variability in the MSVxAS contrast (chosen for its theoretical interest; see Weber et al., 2013; Table 2, IDs 5–14). (3) Confirmatory ROIs: these peaks come from Table 2 of Weber et al. (2015a), from the MSVxAS contrast for the high drug-risk group (Table 2, IDs 15–23). For every ROI irrespective of source, a mask was created by including the central voxel along with all neighboring voxels within a Manhattan distance of 2 voxels (i.e., 10 mm) of the central voxel (total mask volume = 25 voxels; 675 mm^3^). In each ROI, for each pair of participants, we computed similarity as the Euclidean distance between the activity patterns in the MSVxAS contrast statistical parametric map (SPM) within that ROI for that pair of participants (Figures 1A,B).

**TABLE 2:** Location and source of each ROI for *a priori*, exploratory, and functional ROIs.

ID Source	x	Y	z	Region
***A priori* ROIs**				
1 Chua et al., 2009	−8	54	32	BA8/9
2 Ramsay et al., 2013	−46	28	12	BA46
3 Ramsay et al., 2013	−30	12	54	BA6
4 Falk et al., 2016	−4	56	−4	BA10
**Exploratory ROIs**				
5 Exploratory	−36	−38	68	BA5
6 Exploratory	−22	−12	18	Striatum
7 Exploratory	22	−18	−14	BA35
8 Exploratory	46	−50	−10	ITG
9 Exploratory	−12	46	14	BA9
10 Exploratory	−54	4	−26	BA21
11 Exploratory	18	56	4	Forceps Minor
12 Exploratory	−36	−6	16	BA13
13 Exploratory	−36	−2	−14	Inferior Insula
14 Exploratory	−32	12	−42	BA38
**Confirmatory ROIs**				
15 Weber et al., 2015a	−26	−76	−42	Cerebellum
16 Weber et al., 2015a	−44	−60	6	MTG
17 Weber et al., 2015a	−58	−68	0	iLOC
18 Weber et al., 2015a	−38	−84	20	sLOC
19 Weber et al., 2015a	56	4	−18	STG
20 Weber et al., 2015a	6	−52	48	Precuneus
21 Weber et al., 2015a	8	56	38	Frontal Pole
22 Weber et al., 2015a	48	24	28	MFG
23 Weber et al., 2015a	46	−2	28	Precentral Gyrus

*All coordinates given in MNI space. BA, Brodmann area; ITG, inferior temporal gyrus; MTG, middle temporal gyrus; iLOC, inferior lateral occipital cortex; sLOC, superior lateral occipital cortex; STG, superior temporal gyrus; MFG, middle frontal gyrus.*

In addition to MSVxAS SPM similarity, which we take as our dependent variable, we quantified individual differences along a number of other dimensions that might explain this MSVxAS SPM similarity. These dimensions are partitioned into three broad groups: neural measures, other intrinsic measures, and PSA-related measures.

#### Neural Measures

Our neural measures include structural and functional measures of similarity, at both the whole-brain (wbFxnSim) and ROI level. It is important to point out that the whole-brain measures of similarity ostensibly serves as a control by which each ROI might be meaningfully compared. The structural measures are derived from a probabilistic segmentation of each participant’s anatomical scan, while the functional measures come from SPMs of the activity related to the two different control conditions (ROIneur) shown in the PSA experiment (scrambled videos as well as a blank screen). Both structural and functional pattern similarity (Euclidean distance between vectorized maps) at the same level (whole-brain with whole-brain, ROI with ROI) were included as explanatory variables. Additionally, for each of the ROI-specific analyses, the whole-brain MSVxAS SPM similarities were included as an additional explanatory variable, increasing ROI specificity by preventing brainwide differences from driving relationships between MSVxAS SPM similarity within any given ROI and other variables (Figures 1A,B).

#### Intrinsic Measures

The intrinsic measures we considered were participants’ sensation seeking trait, marijuana risk, and study compliance (see section “Intrinsic Measures” above). Between-participant similarities were operationalized as the absolute value of the difference between values for each pair of participants (Figure 1C). Note that although some studies report that treating measures based on multiple items as multivariate and computing a distance in higher-dimensional space yields advantages over using a summary measure (e.g., Chen et al., 2020), in this case, because each measure was theoretically univariate, we opted for the more straightforward summary-score absolute difference.

#### PSA Measures

Lastly, as PSA-related measures we considered participants’ responses to the videos along a number of dimensions, including thought valence, ad liking, positivity/negativity, as well as pAS and pMSV (see section “Participant PSA Evaluations” above), all of which were again converted into between-participant similarities (Figure 1C). In this case, in contrast to the intrinsic measures, the measures are meaningfully multidimensional—e.g., one summary “positive” score (which itself combines across several measures that all conceptually measure “positivity”) for each of 32 videos. Therefore, similarity between individuals was computed in three ways: as the Pearson correlation between each individuals’ 32-video vector; as the absolute difference between the mean across all 32 videos for each participant; and as the absolute difference between the standard deviation across all 32 videos for each participant. Note that although this results in three measures of similarity for each participant pair, rather than the one that would result from a simpler summary-based similarity, it still represents a considerable dimensionality reduction compared to the underlying data (for instance, for the variable *neg*, there were 7 ratings for each of the 32 videos, for a total of 224 values for a single individual).

#### Regression Model

To relate these explanatory variables to our outcome measures, for each ROI, we regressed each MSVxAS SPM similarity measure on the full complement of neural, intrinsic, and PSA-related measures (Figure 1D). We also examined three other sources of variance. The first, in line with previous studies using this same similarity-based individual differences approach (Miller et al., 2012), was a set of participant-specific intercepts, which we refer to as “Unexplained” variance—in other words, unexplained by the set of variables we have included, but in principle explainable on the basis of other (unknown) individual difference factors. The second, which we refer to as “Shared Variance,” is not a separate predictor at all, but rather captures the variance explained in the full model that is not explained by any unique variable or set of variables—in other words, the variance that is explained in the subspace defined by the covariance amongst the other predictors. We have split out this source of variance because it is part of the overall variance explained by the model, but previous studies have treated all variance beyond the sum of the unique variances explained by each variable as unexplainable. Finally, as described above, the model also included a whole-brain functional similarity (wbFxnSim) term, which we treat as a control. Specifically, this term acts as a stand-in for all possible control regions since it is orthogonal to all other explanatory variables in the model.

In order to assign credit to each original predictor variable (some of which were operationalized in the model using multiple regressors), we used a round-robin regression approach—that is, for each original group of regressors, we compared the *R*^2^ of the full model^3^ with the *R*^2^ of a reduced model that excluded those variables, and took the Δ*R*^2^ as our measure of interest. We converted these *R*^2^ to (pseudo) *p*-values using a novel constrained bootstrap approach that takes the non-independence of the similarity values (because any given subject contributes *N*≠1 similarities to every regressor, along with the regressand) into account, which we refer to as *p*^∗^-values to denote the result of this novel approach. We treat these *p*^∗^-values as reflecting the strength of evidence that a particular ΔR^2^ is not due to, e.g., non-independence amongst the similarity values. To simplify visualization of the results, we grouped regions together based on their *p*^∗^-values using hierarchical clustering (Ward’s criterion; Murtagh and Legendre, 2014) and summarized per-group *R*^2^-values using *maximum a posteriori* (MAP) estimates for each group. These MAP estimates are also used only for visualization, to capture the natural intuition that larger groups, along with more homogeneous groups, should produce means further from zero (to the degree supported by the evidence).

## Results

A fundamental goal of this study is to characterize neural responses that are, broadly, sensitive to message tailoring. To that end, we have identified neural ROIs drawn from: the past literature (referred to as *a priori* ROIs), our own previous GLM-based investigations into the MSVxAS contrast on this dataset (referred to as confirmatory ROIs), and a set of ROIs that showed considerable between-participant variability in the MSVxAS contrast (referred to as exploratory ROIs). We utilized a multidimensional similarity based approach where we regressed pairwise similarities on a variety of neural, intrinsic, and PSA-related measures on pairwise neural similarities in the *a priori*, confirmatory, and exploratory ROIs. In what follows, we describe the results of this analysis.

**FIGURE 2:**
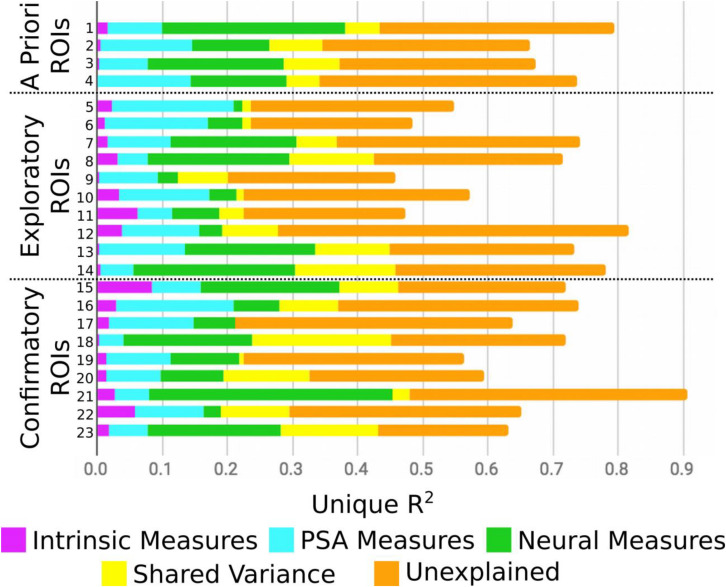
Results from the round-robin regression, split by confirmatory, exploratory, *a priori* ROIs. ROI key corresponds to Table 2.

**FIGURE 3:**
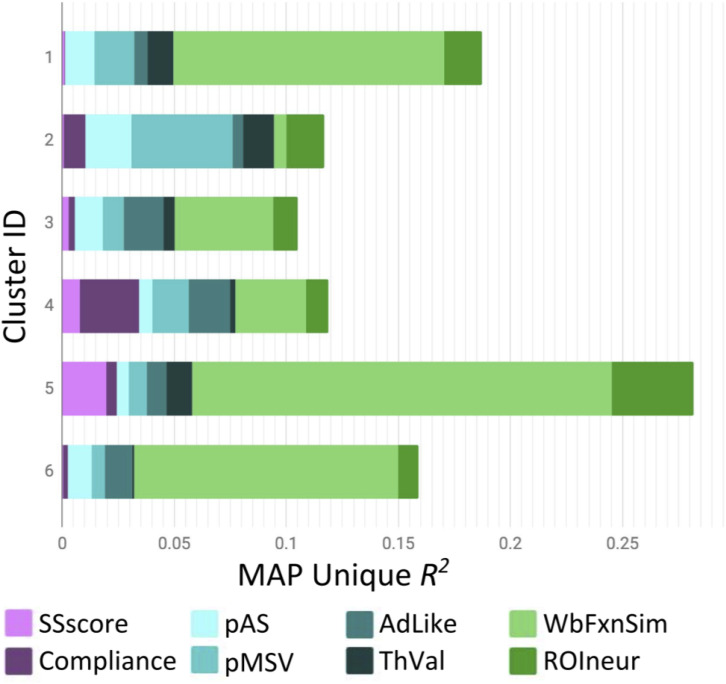
MAP-estimated mean variance accounted for by each variable in each of the six ROI groups identified by Ward clustering. Note that the bars do not sum to 1 because the MAP estimation procedure shrinks estimates toward 0 proportional to the strength of evidence (see also, Table 3). wbFxnSim, whole brain functional similarity for the MSVxAS contrast; pMSV, perceived message sensation value; pAS, perceived argument strength; ThVal, thought valence; ROIneur, region of interest control condition similarity and anatomical similarity; SSscore, sensation seeking score; Compliance, self-reported engagement with the study. Note that this figure is based on clustering of (*z*-transformed) p*-values, but shows group profiles in terms of percent variance accounted for by each variable.

Figure 2 presents the results of the round-robin regressions for each of our 23 ROIs. In order to reduce dimensionality, these results were organized according to the groups discovered by the Ward clustering procedure^4^. To simplify (and at the same time, enrich) the results regarding strength of evidence presented in Table 3, Figure 3 visualizes the MAP estimate of the percent variance accounted for by each variable within each group. A number of patterns emerge. Most notably, relative to other cluster groups, Group 2 evinces a large contribution of similarity in pMSV (4.5% versus an average of 1.1%). As a reminder, this means that above and beyond all other variables, controlling for whole-brain changes, and shrinking the estimate toward 0 in inverse proportion to the strength of the evidence, the similarity between pairs of individuals in terms of their pMSV ratings of the 32 PSAs explained 4.5% of the variance in the similarity between individuals in the patterns of MSVxAS activity within the three areas that make up this cluster. Group 5 meanwhile shows large contributions from the whole-brain MSVxAS similarity (wbFxnSim; 18.7% versus an average of 6.4%), neural similarity in terms of structure and control activity (ROIneur; 3.6% versus an average of 1.3%), and similarity of sensation seeking score (SSscore; 2.0% versus an average of 0.3%). Group 1, by comparison, shows the second largest contribution of wbFxnSim, but also pMSV, pAS, and ThVal.

**TABLE 3:** Variables with a high strength of evidence (p* < 0.05, one-tailed, uncorrected; as described in the text, this is meant only for dimensionality reduction for clustering, rather than drawing inferences) for each ROI, along with the group to which each ROI was assigned in the Ward clustering analysis (see also Figure 3).

ID	Group	Variable 1	Variable 2	Variable 3
1	1	wbFxnSim	—	—
2	1	wbFxnSim*	—	—
4	1	wbFxnSim*	—	—
9	1	wbFxnSim*	pMSV	—
11	1	wbFxnSim	pMSV	—
14	1	wbFxnSim	—	—
15	1	wbFxnSim	pAS	—
21	1	wbFxnSim*	—	—
22	1	wbFxnSim	ThVal*	—
3	2	pMSV	—	—
16	2	pMSV	—	—
23	2	pMSV	—	—
5	3	ROIneur	—	—
12	3	—	—	—
19	3	AdLike	—	—
6	4	Compliance	pMSV	—
17	4	Compliance*	wbFxnSim	—
18	4	Compliance*	—	—
7	5	wbFxnSim**	ROIneur**	—
13	5	wbFxnSim*	SSscore	—
20	5	wbFxnSim	SSscore	ROIneur
8	6	wbFxnSim**	—	—
10	6	wbFxnSim**	—	—

wbFxnSim, whole brain functional similarity for the MSVxAS contrast; pMSV, perceived message sensation value; pAS, perceived argument strength; ThVal, thought valence; ROIneur, region of interest control condition similarity and anatomical similarity; SSscore, sensation seeking score; Compliance, self-reported engagement with the study. *p* < 0.01, **p* < 0.001.

**FIGURE 4:**
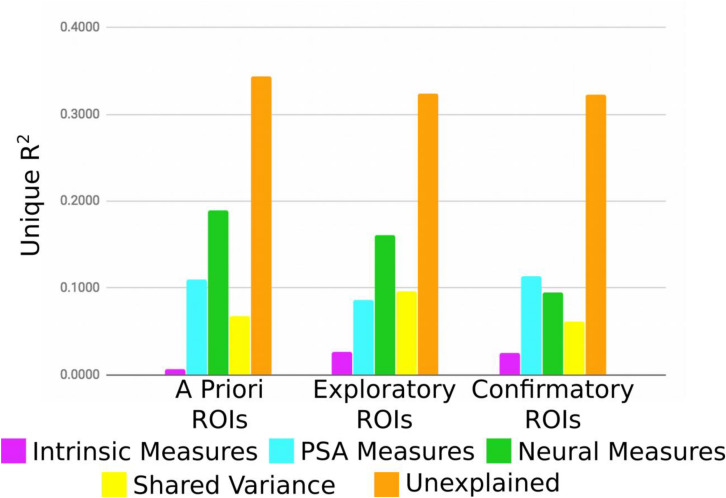
Average unique variance explained for each group of ROIs for each individual difference measure.

As an alternate way of evaluating the analysis, the variance explained for each group of ROIs (*a priori*, exploratory, confirmatory) was averaged without shrinkage for each category of individual difference measure (Figure 4). For *a priori* and exploratory ROIs, neural measures explain the most variance, even after controlling for unexplained sources of variation. Interestingly, PSA measures account for considerable variance across all three ROI groups. Somewhat surprisingly, intrinsic measures explained comparably little variance, regardless of ROI group. However, it is important to point out that according to our novel p^∗^-values, and exactly analogous to a typical OLS context, variables with large amounts of explained variance (or large betas) do not necessarily correspond to those for which there is the strongest evidence that the true amount of explained variance (beta value) is non-zero.

**FIGURE 5:**
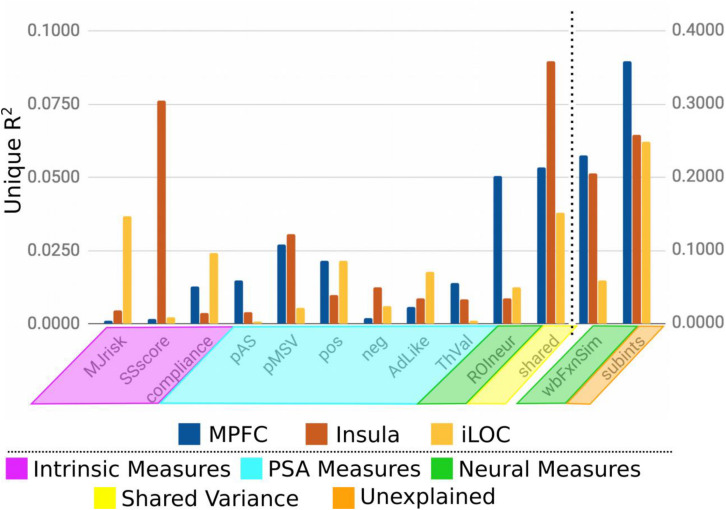
Unique variance explained by each individual difference measure for the MPFC, Insula, and iLOC.

To further examine these results and to more clearly demonstrate broad trends in ROI group differences revealed by our analytical approach, we selected three representative regions (Figure 5), one each from each of the groups of ROIs: MPFC (*a priori* group, from Falk et al., 2016), insula (exploratory group), and inferior lateral occipital cortex (iLOC; confirmatory group). Here we see that each region (and by extension, to varying degrees, each group of ROIs) is particularly sensitive to a number of theoretically relevant individual difference variables. For instance, the similarities on participants’ sensation seeking traits explain considerable variance in MSVxAS similarities within the insula. By comparison, similarities in marijuana risk more prominently account for MSVxAS similarities within the iLOC. Variance in the MSVxAS contrast within the MPFC was not strongly explained by some of the intrinsic or PSA-related individual difference variables we measured here, although it was explained to a higher degree by measures of neural similarity, as well as unspecified individual difference factors not among those we included here (the “Unexplained” variance visible in Figure 5).

## Discussion

In this study, we used a multi-dimensional similarity-based approach for characterizing how a number of brain regions, chosen in several distinct ways, are sensitive to a variety of individual difference factors. We applied this approach to PSAs that were systematically varied in terms of MSV and AS. Previous research has shown that MSV and AS interact to shape persuasive message processing and subsequent behavioral (Petty and Cacioppo, 1984, 1986; Weber et al., 2013) and neural (Weber et al., 2015a; Huskey et al., 2017) outcomes. We show that brain structures across *a priori*, confirmatory, and exploratory ROIs demonstrate different response profiles to intrinsic, PSA-specific, and neural individual difference measures. We now turn to a discussion of these results in terms of their implications for neural models of persuasion, prevention neuroscience and message tailoring, and for methodological advancements.

### Implications for Neural Models of Persuasion

Two important yet unresolved questions in the persuasion neuroscience literature are: (1) what are the relevant structure-function mappings in persuasive message processing, and (2) how selective are these mappings to the putative processes of interest (Huskey et al., 2020). On one hand, a small handful of structures including the vMPFC and VS have been strongly implicated in persuasive message processing as well as in message tailoring (Tompson et al., 2015; Falk and Scholz, 2018) and neural activity in these structures has been repeatedly shown to predict persuasive message outcomes above and beyond what is capable with more traditional measures (Falk et al., 2012, 2016; Pegors et al., 2017; Cooper et al., 2018; Doré et al., 2019).

On the other hand, it has also been demonstrated that a diversity of neural structures (in addition to vMPFC and VS) are recruited during persuasive message processing (e.g., Falk et al., 2010; Doré et al., 2019) and that these structures are sensitive to a number of theoretically relevant moderators (Weber et al., 2015a; Huskey et al., 2017). In fact, several review articles now highlight the diversity of neural structures that are implicated in persuasive message processing (Kaye et al., 2016; Cacioppo et al., 2017). While reiterating that our results are data driven, they come from theoretically informed linkages between intrinsic, PSA, and neural measures. Our results thus provide initial evidence on some of the above questions, which will need to be confirmed with careful forward inference designed to test specific structure/function relationships identified here.

For instance, our results suggest that the MPFC seems rather insensitive to some of the intrinsic (e.g., MJrisk, SSscore) and PSA-related (e.g., neg; AdLike) individual difference variables we measured in this study, and comparably more sensitive to neural (structural) individual differences in participants. Although, it is worth pointing out that unmeasured subject-specific factors (subints) still explain over 35% of the variance in this structure, suggesting that other (unmeasured) variables can explain MSVxAS activity pattern similarities in this structure. As such, the individual difference variables we measured are not suitable as candidates for optimizing message tailoring in the MPFC, but other potential candidates remain (Tompson et al., 2015). In contrast, other regions, including those in our clusters 2, 4, and 5, are relatively more affected by the intrinsic and many of the PSA-related factors we measured, and would likely be regions in brain-as-predictors designs that are more sensitive to the individually varying traits and message perceptions of target audiences.

We now turn briefly to a more detailed evaluation of the results, organized by our three ROI groups (*a priori*, confirmatory, exploratory) before turning to a more general treatment of the methodological implications and limitations of our study.

#### *A priori* ROIs

In this study, we selected four ROIs that have been commonly implicated in persuasive message processing by a diversity of scientific teams. The BA6 and BA46 from Ramsay et al. (2013), the BA8/9/MPFC from Chua et al. (2009), and the BA10/vMPFC from Falk et al. (2016). Notably, the previous two structures have been implicated in value computations during persuasive message processing (Falk and Scholz, 2018) and are implicated as target structures for investigating message tailoring (Tompson et al., 2015). Taken as a group, our results suggest that these regions vary in ways that are in principle explainable on the basis of individual difference factors—very weakly with respect to the intrinsic measures we examined, moderately with the PSA-related measures on which we focused, but fairly strongly as a function of the non-specific participant intercepts, suggesting that some other (as-yet unknown) variable or combination of variables may in fact explain a substantial proportion of the overall variance in MSVxAS activity within these regions.

With our current approach, trying to distinguish further beyond the individual ROIs that constitute this group—which, to be clear, are theoretically heterogeneous and come from prior studies on a number of distinct phenomena—would be overly speculative. We can note, however, two other broad patterns. The first is that these ROIs are organized into two distinct clusters, three in the large and somewhat non-specific cluster 1, which is dominated by the explanatory power of whole-brain MSVxAS similarity (on which we will say more shortly), and the fourth (BA6 from Ramsay et al., 2013) into cluster 2, which is driven by pMSV. Thus, our clustering offers some evidence of functional fractionation amongst these four *a priori* ROIs; we suggest future research in particular might explore the pMSV influence observed in this BA6 ROI further.

The second broad pattern in this group of ROIs, and in particular the three aside from BA6, is the strong influence of whole-brain MSVxAS similarity. On average within these three ROIs, this variable uniquely accounted for 17.1% of the overall variance, compared with an average of 10.2% for all of the other ROIs. This result lends itself to three plausible (though not necessarily equally so) interpretations: either these ROIs are non-specific and so densely interconnected with the rest of the brain that activity changes across the whole brain drive downstream changes in these regions; or, these ROIs act as hub/control regions, such that activity in these regions drives whole-brain patterns of activity. A third way of viewing these results, which is compatible with either of the first two, is that the whole-brain MSVxAS activity pattern can be seen as a sort of “neural fingerprint” reflecting holistically how a specific individual is affected by these two variables. In this view, these ROIs can be seen as a lower-dimensional biomarker of a whole-brain pattern.

However one interprets the nature of this relationship between whole-brain and within-ROI patterns of MSVxAS similarity, the key point is that scholars who investigate these regions should be aware of this linkage—in other words, it appears that each region is integrated into a broader (possibly brain-wide) network and may therefore reflect myriad inputs and outputs. Future research may be able to elucidate whether these regions exhibit predictive utility because of this correspondence to whole-brain activity, or in spite of it.

#### Confirmatory ROIS

In this study, we also selected a number of ROIs based on our previous group-level analysis on the same dataset (Weber et al., 2015a). These were ROIs that were implicated in persuasive message processing, for the MSVxAS contrast (the interaction of two objective, and theoretically relevant message characteristics), among high drug-risk participants (as indicator of high message involvement). In this previous research, we identified these structures as the neural correlates of counterarguing. Including these structures in this individual differences analysis allows us to examine questions related to structure/function selectivity. Beginning first with the aggregate pattern of results for the full set of ROIs, we see a result that is broadly similar to that seen for the *a priori* ROIs, but with two key differences: first, these ROIs show a slightly stronger role for intrinsic variables in driving within-ROI MSVxAS similarity (a still-small 2.5%, but much more than the 0.7% average in the *a priori* ROIs); and second, they show a substantially lower influence of other neural sources (9.5% variance uniquely explained by neural similarity measures versus 18.9% on average across *a priori* ROIs).

It might be surprising that the intrinsic and PSA-related variables do not emerge as even stronger sources of explained variance, given that these measures are theoretically relevant either in understanding what motivations and reactions a viewer might have in general in this task (i.e., as a function of intrinsic variables) or in measuring how they *perceived* the two objective message characteristics that constitute the MSVxAS contrast (i.e., pMSV and pAS). However, further consideration suggests that this result should not, in fact, be surprising. First, because these regions were selected, at least in part, on the basis of having low inter-individual variability (that is, the denominator of our group-level voxelwise test statistic), which is inherently at odds with an analysis designed to capitalize upon relatively high levels of inter-individual variability (see also, Hedge et al., 2018). Second, because pMSV and pAS are theoretically distinct from the (independently derived) MSV and AS measures that defined our MSVxAS contrast, such that similarity in the perceptions of the former may relate only weakly to similarity in the impact of the latter. And third, because pMSV and pAS are considered separately in this analysis, whereas the contrast map was derived from the interaction between the two—in essence, leaving main effect terms on the perceptual level to try to explain an interaction on the objective message characteristics level. In light of these considerations, it is perhaps surprising that these regions saw over 1/8th of the variance in MSVxAS similarity uniquely explained on the basis of similarity on these individually varying perception variables.

In terms of fractionating these ROIs further and attempting to ascribe function to any of them on the basis of these results, the picture is relatively more complicated than for the *a priori* results. Regions from this group are distributed across five of the six clusters, and make up the majority in two of these (clusters 2 and 4). This does suggest that although previous research (Weber et al., 2015a) demonstrates that these regions all showed an interaction between MSVxAS among high drug-risk participants, our results suggest that these ROIs are functionally heterogeneous. With the caveat that the exact magnitude of any particular variable–ROI relationship should be interpreted with caution in our analysis, we highlight four results below that demonstrate this diversity.

First, the only region of the 23 we examined to show an influence with a notably high strength of evidence of pAS similarity (see Table 3) was amongst this set: in cerebellum, the inter-individual similarity in video-by-video ratings of pAS explained 5.7% of the variance in the MSVxAS activity pattern. Although cerebellum has long been thought of as relegated to simple motor coordination and online error-correction, it has emerged more recently as centrally implicated in a wide range of cognitive functions (e.g., Keren-Happuch et al., 2014; King et al., 2019). According to the recent functional parcelation of King et al. (2019), our specific cerebellar ROI is in a functional cluster dominated by divided attention and active maintenance.

Second, considering all of the relationships between theoretically relevant variables and MSVxAS similarity identified as having a high strength of evidence by our bootstrap-based thresholding approach, two of the three strongest relationships involved members of this group. In both cases, the proportion of variance in MSVxAS similarity explained by similarity on the compliance variable had a high strength of evidence. Although the unique variance explained is small (2.4 and 2.6%, respectively), both inferior and superior lateral occipital cortical ROIs evinced an influence of compliance. Given lateral occipital cortex’s role in visual processing, this may suggest that participants’ subjective motivation during the experimental task modulated perceptual processing in such a way that it impacted the evaluative processing reflected in the MSVxAS contrast. Behavioral evidence using eye-tracking corroborates this interpretation (Stevens et al., 2020).

Lastly, of the three strongest variable-similarity relationships mentioned above, the third also involved a member of this group, namely between thought valence (a common behavioral predictor of message persuasiveness) and MSVxAS similarity within the confirmatory MFG ROI, where thought valence similarity uniquely explained 7.7% of the MSVxAS similarity. This ROI is in the posterolateral aspect of BA9, and has been implicated with language comprehension, semantic processing, and related high-order cognitive functions (Wilson et al., 2008). This ROI is also frequently isolated as a target region in persuasion neuroscience studies with a focus on health-related behaviors and message tailoring. For instance, Ramsay et al. (2013) found this region to be more strongly activated by strong (i.e., persuasive) health messages compared to weak ones, Chen et al. (2018) show MFG activation is greater when viewing e-cigarette advertisements compared to non e-cigarette advertisements, and Chua et al. (2009) found this region to be more active in high-tailored health messages compared to low-tailored ones.

#### Exploratory ROIs

These ROIs were selected on the basis of evincing high inter-individual variability, but without reference to whether that variability was explainable. Therefore, the fact that several of the relationships between MSVxAS similarity and similarity on our other variables of interest emerge as having a high strength of evidence is validation of this general approach to identifying potential ROIs. By definition, due to the variability between individuals, these are areas that are relatively less likely to be observed in a typical group-based GLM analysis. Nonetheless, these results are in line with the claim made by previous scholars (Van Horn et al., 2008; Gabrieli et al., 2015) that such variability does not necessarily reflect only noise, but may reflect meaningful signal. In fact, taking a simple average of the sum of Δ*R*^2^-values associated with the variables that most plausibly relate to the contrast of interest—that is, marijuana risk, sensation seeking, compliance, pAS, and pMSV, all of which might be expected to contribute to the unique activation in the high-risk MSVxAS map from which we took our confirmatory ROIs—the mean for this group of exploratory ROIs narrowly edges out the confirmatory ROIs (7.1–6.7%) and convincingly surpasses the *a priori* ROI average (4.3%). Although this is far from proof that these regions are involved in persuasive processing, either causally or downstream of other regions, it does support the premise that these regions may differ reliably between individuals along theoretically interesting vectors.

Otherwise, as a group, these ROIs show a similar pattern as the other two groups: the neural similarity variables explain an amount of variance in between *a priori* and confirmatory ROIs on average (16.1%), with slightly more of the variance shared amongst the other predictors explaining MSVxAS similarity (9.7% for this group compared to 6.8 and 6.1% for *a priori* and confirmatory, respectively), and a similar amount of explanatory power from unmeasured participant-specific sources (32.4% compared to 34.4 and 32.2%, respectively). As with the confirmatory group of ROIs, the exploratory ROIs are distributed across five of six clusters, and are the majority in three (3, 5, and 6). Thus, as might be expected for a group of ROIs chosen only on the basis of varying widely in terms of MSVxAS activity across participants, these regions appear to be quite heterogeneous, with the most consistent relationship being the strong influence of the whole-brain MSVxAS pattern similarity—although even here, there is a split between those regions where the relationship had a high strength of evidence (7 regions, average Δ*R*^2^ of 16.9%) and those where it did not (3 regions, average Δ*R*^2^ of 4.3%). Finally, although these exploratory ROIs should be seen only as targets for future investigation, we highlight a pair of ROIs that may warrant such attention. In each of the two, MSVxAS similarity relates moderately with pMSV similarity: 5.8% unique variance explained in striatum, and 5.7% unique variance explained in medial BA9.

### Implications for Prevention Neuroscience and Message Tailoring

To return to an issue we touched upon above, the last 10 years of research in persuasion neuroscience have seen the development of an exciting new methodology, namely the brain-as-predictor approach (Falk et al., 2012; Berkman and Falk, 2013). The seminal application of this approach included two compelling results: the first—that neural activity from a small group can be used to predict outcomes in large independent samples (see Figure 1; Falk et al., 2012)—has been written about extensively, and gives the approach its name. No less interesting, however, is the finding that individual-specific brain activity matched the group consensus, and therefore the population prediction, in only 1 out of 3 individuals (see Figure 2; Falk et al., 2012)—in other words, although the modal/plurality response is indeed useful in predicting modal/plurality responses at the population level, this to some degree masks the fact that this rank-ordering of activity reflects a minority within the scanned sample. We suggest, on the basis of a decade of research in cognitive neuroscience, that such variability between individuals represents an opportunity, not a nuisance (Van Horn et al., 2008). By better understanding which factors unique to each individual drive the relationship between messages and brain activity, we may be able to improve out-of-sample prediction as well as message tailoring.

We propose that a hybrid approach, inspired by the analyses conducted here, offers a way forward. In particular, when translating our results into their utility in identifying potential targets for tailoring, one would look for regions with a comparatively high proportion of variance explained by variables of theoretical interest—in this case, intrinsic variables such as marijuana risk and sensation seeking traits or PSA-related variables such as pMSV or pAS, although of course these will differ depending on the theoretical question under investigation—and a correspondingly low proportion of variance explained by factors such as whole-brain pattern similarity for the relevant contrast, or the similarity within the region along non-specific dimensions such as anatomical structure or activity during a control task. In the case of the current analysis, the region that most fits this description is the striatal region from the exploratory group of ROIs, which has modest effects of pMSV and compliance that have a high strength of evidence, with quite low values for neural variables, whether whole-brain or ROI-specific. This suggests that this region is task-relevant in a way that is specific to this region and contrast. We would expect that, were an analysis conducted in order to identify prospective candidate regions that fit this profile of high/low variance explained for theoretical/nuisance variables (e.g., using canonical correlation analysis, see below), the resulting regions would be even more specific, with stronger relationships, and more potential for use as biomarkers in future investigations of tailoring and persuasion.

One last way in which our approach reveals targets that may be ripe for future investigation, and possibly eventually targeting, is through the inclusion of the non-specific subject intercept variables. As we stated above, the variance explained by these variables represents variance that is in-principle explainable, by variables other than those included in our analyses. Thus, an ROI with a high degree of variance accounted for by these intercepts, particularly if the ROI is of interest on theoretical or meta-analytic grounds, deserves extra attention; although it seems unlikely, it may be that a single variable could explain the bulk of this variance, which for the ROIs we examined here ranged from 20% to a staggering 54% of unique variance.

### Methodological Implications

The first implication of our results, which aligns with recommendations that have been growing increasingly strident in the past several years (Turner et al., 2018; Finn et al., 2020), is that when it comes to group-average results in the context of tasks that allow for a wide diversity of responses, researchers should be aware that any activation that is shared across all subjects may be dwarfed by activity that is present only amongst some subset of individuals (see also, Hawco et al., 2020). In this vein, we encourage researchers working in this area to follow the example set by Falk et al. (2012) of reporting not only average results, but something that reveals the underlying variability within the sample. This can also include publication (in Supplementary Materials or public databases, if not in the main text) of unthresholded group SPMs as well as maps showing the correspondence (or lack thereof) across individuals (e.g., using overlap maps, Seghier and Price, 2016).

It is worth pointing out as well that there are untapped dimensions of variance that we did not examine in the present study, but which research using complementary approaches has suggested bear further study. For instance, we reduced the set of 32 30-s PSAs to a single MSVxAS interaction map, by characterizing every video along those two dimensions and averaging across all videos. However, considerable work has demonstrated the power of considering the full temporal extent of the sort of spatiotemporal stimuli we use here (e.g., Hasson et al., 2004), and this concept has previously been extended to consider individual differences (Nguyen et al., 2019; Chen et al., 2020; Finn et al., 2020), and even to jointly consider spatiotemporal patterns (Hyon et al., 2019). As some of these investigations point out, this approach also lends itself naturally to a combination with representational similarity analysis, which can accommodate data from a broad range of sources (Finn et al., 2020). Lastly, approaches based on canonical correlation analysis have also begun to be adapted to the study of individual differences (Biessmann et al., 2014; Wang et al., 2020).

One final consideration, the extent to which structures implicated in our analysis truly belong to the persuasion network is, as of yet, unknown. At a fundamental level, demonstrating this would require different methods that explicitly test the networked functional connections between two or more ROIs. A variety of analytical tools already exist for doing exactly this, including: psychophysiological interaction analysis (Friston et al., 1997), dynamic causal modeling (Friston et al., 2003), and graph-theoretic techniques (Fornito et al., 2016; Bassett and Sporns, 2017; Turner et al., 2019). These techniques have the benefit of explicitly examining a structure’s involvement within a broader constellation of structures. In addition, there is good reason to believe that this approach can further illuminate individual differences (see e.g., Yeo et al., 2015), especially when more classic ROI-based approaches fail (see e.g., Fox et al., 2014). Issues of circularity make it inappropriate to perform a network analysis on this dataset with the structures identified in our similarity analysis (Vul et al., 2009; Weber et al., 2015b). Nevertheless, our study certainly points out candidate ROIs for future confirmatory analysis on new datasets.

### Limitations

As we alluded to, these analyses represent *post hoc* reuse of data originally designed and collected to address a related, but distinct, hypothesis. This practice is commonplace in cognitive neuroscience, and is not inherently problematic, so long as researchers are transparent about the fact, and aware of the consequences this practice has. In this case, we see this work as filling an inductive role—we largely eschew inferential statistics, and where we do consider *p*^∗^-values, we do so in a way that is as conservative as possible, and that recognizes the tentative nature of our findings. We would also reiterate that our results focus on activity patterns associated with one specific theoretically motivated contrast, namely the MSVxAS interaction. It is clear that other contrasts or studies that focused on other contributors to the persuasion process would implicate roles for region-variable combinations that did not appear for this particular contrast.

Future work that is designed to continue in the vein identified here has several strategies available to operate more in the standard tradition of deduction and falsification (e.g., Popper, 1985). For instance, any promising relationships observed here can be operationalized and built into the design of future experiments, most critically, with careful measurement and perhaps even stratified sampling of participants on the basis of individual difference measures of interest. The issue of non-independence which was addressed here by a novel constrained bootstrap technique will also need to be solved more satisfactorily; the approach advocated by Chen et al. (2016, 2017, 2019) is promising, but must be validated in its application in this context. Likewise, the Mantel test (Mantel, 1967) has been applied previously in a similar context (Chen et al., 2020), but has not been thoroughly validated.

## Conclusion

This work demonstrates the importance of considering the correct level of specificity when studying health message processing—in terms of message characteristics as well as audience characteristics—and points the way toward possible updates to the persuasion network. Moreover, these results have the power to positively influence the field of prevention neuroscience. Prediction of health campaign success using a combination of self-report and neural measures has made tremendous progress and shown intriguing results, but is still nowhere near perfect. We argue that variability between individuals represents an opportunity, not a nuisance. By better understanding which message and audience characteristics drive which neural responses to health communication campaigns, we may be able to improve our ability to tailor audience-specific health messages and predict which messages are most likely to result in health attitude and behavior change even more accurately.

## Data Availability Statement

The datasets presented in this study can be found in online repositories. The names of the repository/repositories and accession number(s) can be found below: Open Science Framework (https://osf.io/vmc8e/).

## Ethics Statement

The studies involving human participants were reviewed and approved by the Human Subjects Committee of the University of California, Santa Barbara. The patients/participants provided their written informed consent to participate in this study.

## Author Contributions

RW: idea, study design, and data collection. RH, BT, and RW: concept and analysis, manuscript writing, and methods and materials. RH and BT: figures. All authors contributed to the article and approved the submitted version.

## Conflict of Interest

The authors declare that the research was conducted in the absence of any commercial or financial relationships that could be construed as a potential conflict of interest.

## Footnotes

1 It is important to note that these limitations extend to any fMRI-based research that is organized around group-level analyses; see also Turner et al. (2019) for further discussion of such limitations.

2 https://osf.io/vmc8e/

3 This full model excluded the participant-specific intercepts described above for all regressions except for the one for determining the unique contribution of those intercepts. This is akin to hierarchical regression, and is done to prevent this non-specific variable from absorbing variance that can be explained by the other specific variables.

4 Note: Each analysis was run independently; see Table 2 for ROI labels and Table 3 for the assignment of ROIs to groups.
